# Prognostic impact of eligibility for adjuvant immunotherapy in locally advanced urothelial cancer

**DOI:** 10.1002/bco2.117

**Published:** 2021-10-08

**Authors:** Yuki Miura, Shingo Hatakeyama, Toshikazu Tanaka, Naoki Fujita, Hirotaka Horiguchi, Yoshiharu Okuyama, Yuta Kojima, Daisuke Noro, Noriko Tokui, Teppei Okamoto, Hayato Yamamoto, Hiroyuki Ito, Takahiro Yoneyama, Yasuhiro Hashimoto, Chikara Ohyama

**Affiliations:** ^1^ Department of Urology Hirosaki University Graduate School of Medicine Hirosaki Japan; ^2^ Department of Urology Aomori Prefectural Central Hospital Aomori Japan; ^3^ Department of Urology Mutsu General Hospital Mutsu Japan; ^4^ Department of Urology Odate Municipal Hospital Odate Japan; ^5^ Department of Urology Aomori Rosai Hospital Hachinohe Japan; ^6^ Department of Advanced Transplant and Regenerative Medicine Hirosaki University Graduate School of Medicine Hirosaki Japan

**Keywords:** adjuvant immunotherapy, cystectomy, nephroureterectomy, prognosis, urothelial carcinoma

## Abstract

**Objective:**

To evaluate the effect of postoperative pathological findings related to the eligibility of adjuvant immunotherapy on oncologic outcomes in patients with localized and locally advanced muscle‐invasive bladder carcinoma (MIBC) and upper tract urothelial carcinoma (UTUC).

**Patients and methods:**

We retrospectively evaluated 1082 patients treated with radical cystectomy (*n* = 597) and nephroureterectomy (*n* = 485) between January 2000 and April 2021. Patients were divided into two groups: pT3‐4 or pN+ without neoadjuvant chemotherapy and ypT2‐4 or pN+ treated with neoadjuvant chemotherapy (trial‐eligible group) or others (trial‐ineligible group). The primary outcome was the effect of trial eligibility for adjuvant immunotherapy on disease‐free survival (DFS) and overall survival (OS). Secondary outcomes included the additional effect of lymphovascular invasion (LVI) status to the clinical trial criteria on prognosis and a risk model development.

**Results:**

The median ages of the patients were 69 and 72 years in the MIBC and UTUC groups, respectively. Fifty‐two percent of patients met the trial inclusion criteria. Trial eligibility was significantly associated with poor DFS and OS among patients with MIBC and UTUC. LVI‐positive status was significantly associated with poor prognosis among patients in the trial‐eligible group. A very high risk (LVI+ or pN+ among the pT3‐4 or ypT2‐4) was significantly associated with poor prognosis.

**Conclusion:**

A total of 52% of patients were eligible for adjuvant immunotherapy. Trial eligibility was significantly associated with a poor prognosis. LVI+ and pN+ may play a key role in candidate selection for adjuvant immunotherapy.

## 1 INTRODUCTION

Localized or locally advanced urothelial carcinoma (UC) is a life‐threatening disease with a high recurrence and mortality rate (5‐year survival rate: 50%–60%).^1^, ^2^, ^3^ Although radical cystectomy (RC) or nephroureterectomy (RNU) is the standard‐of‐care first‐line treatment, patient prognosis is limited even when using neoadjuvant chemotherapy (NAC) and extended pelvic lymph node dissection.^4^, ^5^, ^6^, ^7^, ^8^, ^9^, ^10^ Adjuvant chemotherapy is an alternative strategy to improve survival, but the administration of toxic chemotherapy in all patients is not feasible because of the advanced age, renal impairment, and frailty in patients with UC. The CheckMate 274 trial demonstrated a benefit in disease‐free survival (DFS) with adjuvant nivolumab therapy in patients at high risk of muscle‐invasive UC and may become a standard of care in the future.^11^ In that study, postoperative pT3‐4/ypT2‐4 or pN+ was used as an inclusion criterion for the high‐risk group, but the validity of this criterion in clinical practice remains unclear. Also, there is an urgent need for the proportion of patients who are eligible for adjuvant immunotherapy in clinical practice.

Conversely, the primary endpoint was not met in the similar IMvigor 010 study, which evaluated the effect of adjuvant atezolizumab after radical surgery.^12^ In that study, the authors found no significant difference in DFS between atezolizumab and observation (median 19.4 vs. 16.6 months, respectively; hazard ratio 0.89; *p* = 0.2446).^12^ Although there is no clear reason for these controversial results, the outcome might have potentially been affected by some key confounding factors. Of the inclusion criteria of those phase III studies, the patient's lymphovascular invasion (LVI) status was not included in the definition of high‐risk disease. Because LVI status is one of the established pathological risk factors for poor prognosis in patients with UC,^13^, ^14^, ^15^, ^16^, ^17^, ^18^, ^19^ we hypothesize that it might play a key role in the selection of potential candidates for adjuvant immunotherapy. Firstly, we evaluate the effect of trial eligibility for adjuvant immunotherapy on prognosis in patients with localized and locally advanced muscle‐invasive bladder carcinoma (MIBC) and upper tract urothelial carcinoma (UTUC) in a real‐world practice. We subsequently evaluate the additional effect of LVI status to the clinical trial criteria on patient prognosis and develop a risk model that includes LVI status.

## 2 MATERIALS AND METHODS

### 2.1 Design and ethics statement

We conducted this retrospective, multicenter study in accordance with the Declaration of Helsinki. The study was approved by the ethics committee of the Hirosaki University School of Medicine (2019–099) and all hospitals in this study. Written consent was not obtained in exchange for public disclosure of study information (opt‐out approach).

### 2.2 Patient selection and demographics

**FIGURE 1:**
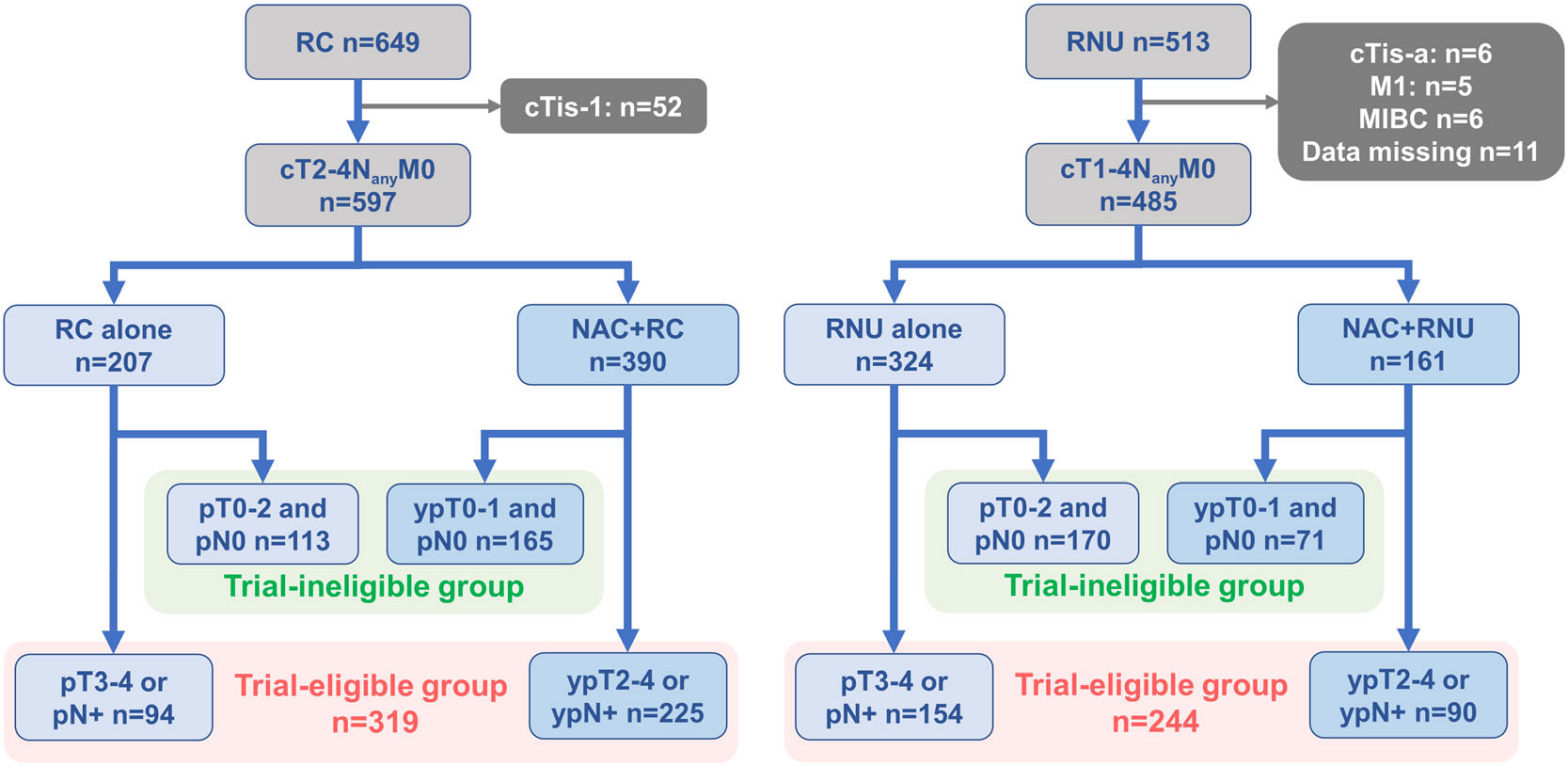
Patient selection. Patient selection for trial‐eligible and ‐ineligible groups treated with radical cystectomy (RC) or nephroureterectomy (RNU). MIBC, muscle‐invasive bladder carcinoma; NAC, neoadjuvant chemotherapy

We retrospectively evaluated 1162 patients with localized or locally advanced UC (MIBC and UTUC) without distant metastasis (M0) who received RC (*n* = 649) and RNU (*n* = 513) between January 2000 and April 2021 at one academic center and five general hospitals. We excluded 52 patients with RC who had cTis‐1 disease (mainly patients with bacillus Calmette‐Guérin unresponsive disease) and 28 patients with UTUC who had cTis‐1, M1 disease, concomitant MIBC, and insufficient clinical data from this study. Finally, we included 597 patients with MIBC and 485 patients with UTUC (Figure 1). The following variables were collected and analyzed: age, sex, Eastern Cooperative Oncology Group performance status (ECOG PS), estimated glomerular filtration rate (eGFR), clinical stage, pathological stage, LVI status, DFS, and overall survival (OS). Tumor stage and grade were stratified by the 8th edition of the TNM classification.^20^

### 2.3 Platinum‐based neoadjuvant or adjuvant chemotherapy

We selected regimens based on our guideline for cisplatin eligibility. Indications for NAC were MIBC ≥T2, UTUC ≥T3, or cN+ disease. In our practice, adjuvant chemotherapy is not routinely administered. Indications for adjuvant chemotherapy include patients with pT4, positive surgical margin, or pN+ who were not treated with NAC. We administered two or three cycles of adjuvant chemotherapy in selected patients with feasible postoperative status for toxic chemotherapy. Patients received either gemcitabine plus cisplatin, gemcitabine plus carboplatin, or methotrexate, vinblastine, doxorubicin, and cisplatin.^2^, ^21^ Cycles were repeated every 21 days for up to four cycles in the NAC setting. For cisplatin‐ineligible patients, we designed a short‐term carboplatin‐based NAC followed by immediate surgery within 90 days from diagnosis to minimize the potential disadvantage.^6^, ^22^, ^23^ Cisplatin‐ineligible patients can take advantage of the waiting time for surgery with this strategy.

### 2.4 Eligibility of cisplatin‐based chemotherapy

Because of the population difference, we used the modified cisplatin‐ineligible criteria of Galsky et al.^24^ Using the original criteria, a patient defined as cisplatin ineligible would meet at least one of the following criteria: ECOG PS > 1, creatinine clearance <60 ml/min or eGFR <50 ml/min/1.73 m^2^, grade >1 hearing loss, grade >1 neuropathy, and/or New York Heart Association (NYHA) Class III heart failure. In addition, we defined the marginal criteria as being ECOG PS 1, eGFR 50–60 ml/min/1.73 m^2^, NYHA Class II heart failure, and age >80 years. Patients with two or more marginal factors (such as ECOG PS 1 and eGFR 55 ml/min/1.73 m^2^) were classified as a cisplatin ineligible.

### 2.5 Surgical procedures

RC or RNU was performed using the previously described basic technique.^25^, ^26^ Briefly, patients with MIBC underwent RC, urinary diversion (orthotopic ileal neobladder construction, ileal conduit diversion, and cutaneous ureterostomy) and standard pelvic lymph node dissection. In patients with UTUC, we performed open or laparoscopic RNU, which includes the removal of the kidney, ureter, and ipsilateral bladder cuff. We managed the distal ureter using the extravesical approach. We performed regional lymph node dissection only when an obvious lesion was observed on imaging study findings.^27^

### 2.6 Outcomes

We divided the patients into two groups: pT3‐4 or pN+ without NAC and ypT2‐4 or pN+ treated with NAC (trial‐eligible group) or others (trial‐ineligible group). A DFS event was defined as the length of time from primary treatment to recurrence or death. An OS event was defined as the length of time after primary treatment to last follow‐up or any cause of death. The primary outcome was the effect of trial eligibility for adjuvant immunotherapy on the DFS and OS. In the case of UTUC, superficial recurrences of bladder tumors were not included in the visceral DFS. Secondary outcomes included the additional effect of LVI status to the clinical trial criteria on prognosis, risk model development, and a comparison of the Harrell's concordance index (c‐index)^28^ and net benefit^29^ between the base model (pT3‐4/ypT2‐4 or pN+) and the LVI model (pT3‐4/ypT2‐4 and pN+ or LVI+) in patients with MIBC and UTUC.

### 2.7 Statistical analyses

We performed statistical analyses by using BellCurve for Excel 3.10 (Social Survey Research Information Co., Ltd., Tokyo, Japan), GraphPad Prism 7.00 (GraphPad Software, San Diego, CA, USA), and R: 4.0.2, A Language and Environment for Statistical Computing (The R Foundation, Vienna, Austria). We tested the intergroup difference using the Student's *t* test or Mann–Whitney *U* test. We used Fisher's exact test or *χ* ^2^ test to compare categorical variables. Quantitative variables were expressed as means with standard deviations or medians with interquartile ranges. The rate of OS from the initial treatment until death was estimated using the log‐rank test. To investigate the effect of LVI status on the DFS and OS, we used multivariable Cox regression proportional hazards model. Hazard ratio with 95% confidence interval were calculated after controlling for potential confounders, including patient age, sex, ECOG PS, tumor type (UTUC), NAC, and pT and pN stage.

## 3 RESULTS

### 3.1 Baseline characteristics

The median ages of the patients with MIBC and UTUC were 69 and 71 years, respectively. Table 1 presents the baseline characteristics of the patients. The numbers of patients who were trial‐eligible and ineligible were 278 and 319 in the MIBC group and 241 and 244 in the UTUC group, respectively. The proportions of patients with trial eligibility for adjuvant immunotherapy were 54% and 50% in the MIBC and UTUC groups, respectively. Of 319 patients with MIBC in the trial‐eligible group, 225 (71%) received NAC. Of 214 patients with UTUC in the trial‐eligible group, 90 (37%) received NAC (Figure 1).

**TABLE 1:** Background of patients

	MIBC			UTUC		
	Trial ineligible	Trial eligible	*p* value	Trial ineligible	Trial eligible	*p* value
*n*	278	319		241	244	
Median age, years (IQR)	69 (62–74)	70 (63–75)	0.225	72 (65–77)	73 (65–79)	0.071
Sex (male), *n*	239 (86%)	237 (74%)	<0.001	164 (68%)	167 (68%)	0.829
ECOG PS >0, *n*	6 (2%)	12 (4%)	0.196	21 (9%)	32 (13%)	0.145
Hypertension (HTN), *n*	79 (28%)	107 (34%)	0.177	116 (48%)	111 (45%)	0.560
Diabetes mellitus (DM), *n*	41 (15%)	44 (14%)	0.740	51 (21%)	34 (14%)	0.042
Cardiovascular disease (CVD), *n*	25 (9%)	48 (15%)	0.025	32 (13%)	34 (14%)	0.924
Chronic kidney disease (CKD) Stage 3–4	79 (28%)	131(43%)	<0.001	145 (60%)	192 (79%)	<0.001
Neoadjuvant chemotherapy (NAC), *n*	165 (59%)	225 (71%)	0.004	71 (29%)	90 (37%)	0.084
Cisplatin‐based regimens, *n*	53 (32%)	41 (18%)	0.002	26 (11%)	13 (5%)	0.030
Clinical stage, *n*						
cT3 or 4	97(35%)	212(66%)	<0.001	91 (38%)	197 (81%)	<0.001
cN+	18 (6%)	46 (14%)	0.002	7 (3%)	34 (12%)	<0.001
Surgical outcomes						
Variant histology, *n*	15 (5.4%)	23 (7.2%)		7 (2.9%)	12 (4.9%)	
Laparoscopic surgery, *n*	0 (0%)	0 (0%)		35 (15%)	36 (15%)	1.000
Robotic surgery, *n*	32 (12%)	22 (7%)	0.062	0 (0%)	0 (0%)	
Urinary diversion (neobladder), *n*	191 (69%)	137 (43%)	<0.001	0 (0%)	0 (0%)	
Pathological outcomes, *n*						
Tumor grade (high)	175 (63%)	315 (99%)	<0.001	231 (88%)	243 (99%)	<0.001
pT0	99 (36%)	3 (1%)	<0.001	27 (11%)	0 (0%)	
pT3 or 4	0 (0%)	309 (97%)		0 (0%)	203 (83%)	
pN+	0 (0%)	71 (22%)		0 (0%)	28 (11%)	
Lymphovascular invasion (LVI+)	31 (11%)	174 (55%)	<0.001	27 (11%)	117 (48%)	<0.001
Adjuvant chemotherapy, *n*	3 (1.1%)	1 (0.3%)		2 (0.8%)	8 (3.3%)	0.106
Tumor recurrence, *n*	41 (15%)	154 (48%)	<0.001	29 (12%)	110 (45%)	<0.001
Deceased, *n*	77 (28%)	161 (50%)	<0.001	39 (16%)	107 (44%)	<0.001
Median follow‐up, months (IQR)	67 (34–107)	32 (12–74)		54 (29–83)	42 (19–70)	

Abbreviations: ECOG PS, Eastern Cooperative Oncology Group performance status; IQR, interquartile range; LVI, lymphovascular invasion; MIBC, muscle‐invasive bladder carcinoma; UTUC, upper tract urothelial carcinoma.

### 3.2 Primary outcome

**FIGURE 2:**
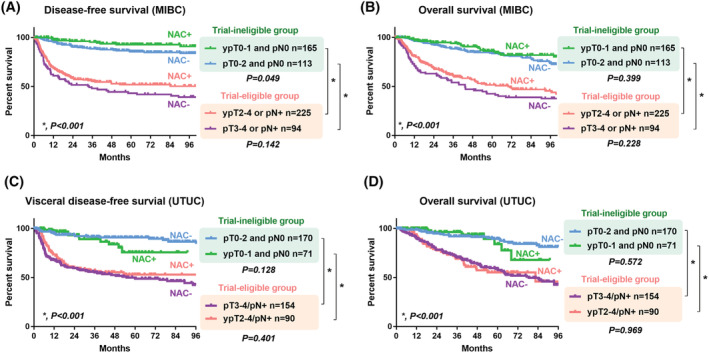
Primary outcomes. (A) Comparison of disease‐free survival between the trial‐eligible and ‐ineligible groups in patients with MIBC. (B) Comparison of overall survival between the trial‐eligible and ‐ineligible groups in patients with MIBC. (C) Comparison of disease‐free survival between the trial‐eligible and ‐ineligible groups in patients with UTUC. (D) Comparison of overall survival between the trial‐eligible and ‐ineligible groups in patients with UTUC. *p < 0.001. MIBC, muscle‐invasive bladder carcinoma; UTUC, upper tract urothelial carcinoma

We found a significant difference in DFS and OS between the trial‐eligible and ‐ineligible groups among patients with MIBC (Figure 2A, B) and UTUC (Figure 2C,D). On the other hand, there was no significant difference in DFS (*p* = 0.142, Figure 2A) or OS (*p* = 0.228, Figure 2B) in the trial‐eligible group among patients with MIBC. Visceral DFS (*p* = 0.401, Figure 2C) and OS (*p* = 0.969, Figure 2D) were not significantly different in the trial‐eligible group of patients with UTUC. The supplement figures present the unadjusted outcomes of DFS and OS stratified by (y)pT0‐1, 2, 3‐4, and pN stage in patients with MIBC (Figure S1) and UTUC (Figure S2).

### 3.3 Secondary outcomes

Multivariable Cox regression proportional hazards model showed a significant effect of LVI and pN+ for DFS and OS in both patients with MIBC and patients with UTUC (Table 2). In the trial‐eligible group of MIBC patients, 41% and 18% of patients were LVI+ or pN+ and LVI+ and pN+, respectively (Figure S3A). The duration of DFS was significantly shorter in patients with LVI+ (*p* < 0.001, Figure S3B) or pN+ (*p* < 0.001, Figure S3C). In the trial‐eligible group of patients with UTUC, 44% and 7.8% of patients were LVI+ or pN+ and LVI+ and PN+, respectively (Figure S3D). The duration of DSF was significantly shorter in patients with LVI+ (*p* < 0.001, Figure S3E) or pN+ (*p* < 0.001, Figure S3F).

**TABLE 2:** Multivariable Cox regression analysis for the trial‐eligible group

		DFS			OS		
		*p* value	HR	95% CI	*p* value	HR	95% CI
MIBC							
Age	Continuous	0.021	1.02	1.00–1.04	<0.001	1.04	1.02–1.06
Sex	Male	0.890	1.03	0.72–1.47	0.641	1.09	0.77–1.54
ECOG‐PS	>0	0.919	1.05	0.45–2.45	0.329	0.63	0.25–1.59
NAC	Underwent	0.494	1.14	0.78–1.65	0.862	1.03	0.71–1.49
pT	2–4	0.172	1.17	0.93–1.47	0.881	0.98	0.79–1.22
pN	Positive	<0.001	2.04	1.42–2.93	0.004	1.71	1.19–2.46
LVI	Positive	0.001	1.87	1.29–2.70	0.004	1.71	1.19–2.44
UTUC							
Age	Continuous	0.384	1.01	0.99–1.03	0.025	1.03	1.00–1.05
Sex	Male	0.215	0.77	0.52–1.16	0.837	0.96	0.63–1.45
ECOG‐PS	>1	0.882	1.07	0.43–2.66	0.545	1.33	0.53–3.34
NAC	Underwent	0.316	1.25	0.81–1.92	0.211	1.34	0.85–2.12
pT	2–4	0.029	1.61	1.05–2.48	0.108	1.45	0.92–2.30
pN	Positive	0.001	2.32	1.43–3.77	0.029	1.78	1.06–2.98
LVI	Positive	<0.001	2.17	1.42–3.32	0.049	1.53	1.00–2.34

Abbreviations: CI, confidence interval; DFS, disease‐free survival; ECOG PS, Eastern Cooperative Oncology Group performance status; HR, hazard ratio; IQR, interquartile range; LVI, lymphovascular invasion; MIBC, muscle‐invasive bladder carcinoma; NAC, neoadjuvant chemotherapy; OS, overall survival; UTUC, upper tract urothelial carcinoma.

### 3.4 Development of a risk model

**FIGURE 3:**
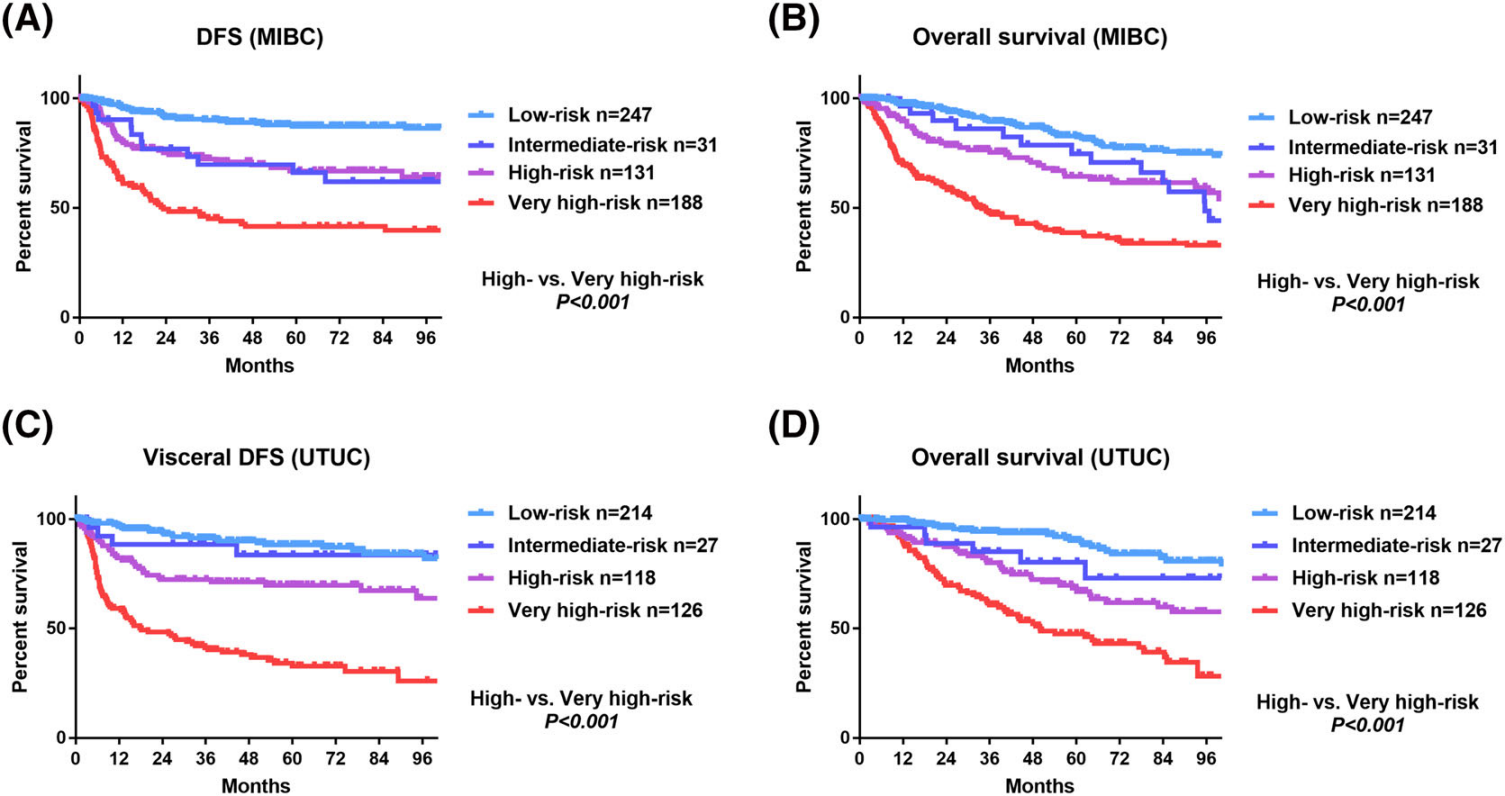
Secondary outcomes (prognostic impact of LVI model). (A) Disease‐free survival of patients with MIBC using the LVI model. (B) Overall survival of patients with MIBC using the LVI model. (C) Disease‐free survival of patients with UTUC using the LVI model. (D) Overall survival of patients with UTUC using the LVI model. DFS, disease‐free survival; LVI, lymphovascular invasion MIBC, muscle‐invasive bladder carcinoma; UTUC, upper tract urothelial carcinoma

**FIGURE 4:**
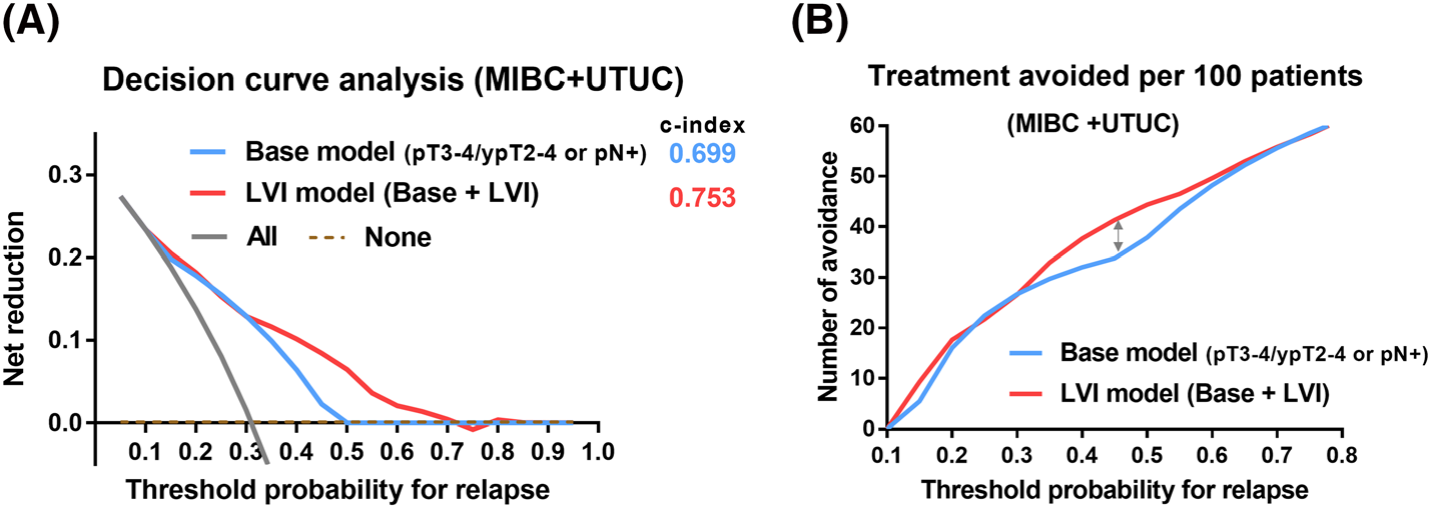
Secondary outcomes (decision curve analysis). (A) Comparison of Harrell's concordance index (c‐index) and net benefit between the base model (pT3‐4/ypT2‐4 or pN+) and the LVI model (pT3‐4/ypT2‐4 and pN+ or LVI+) in patients with MIBC and UTUC. (B) Number of interventions avoided in the base and LVI models. DFS, disease‐free survival; LVI, lymphovascular invasion; MIBC, muscle‐invasive bladder carcinoma; UTUC, upper tract urothelial carcinoma

Accordingly, we developed the LVI model using the pT/ypT stage as well as pN and LVI status and stratified patients into the following four groups: low‐risk (pT0‐2 or ypT0‐1, and pN0 and LVI−), intermediate‐risk (pT0‐2 or ypT0‐1, and pN0 and LVI+), high‐risk (pT3‐4 or ypT2‐4, and pN0 and LVI−), and very high‐risk (pT3‐4 or ypT2‐4, and pN+ or LVI+) (Table 3). There was a significant difference in DFS (*p* < 0.001, Figure 3A) and OS (*p* < 0.001, Figure 3B) between the high‐risk and very high‐risk groups in the trial‐eligible group of MIBC. Similarly, we observed a significant difference in DFS (*p* < 0.001, Figure 3C) and OS (*p* < 0.001, Figure 3D) between the high‐risk and very high‐risk groups in the trial‐eligible group of UTUC. The c‐index of the LVI model for DFS was higher than that in the base model (0.753 vs. 0.6994; Figure 4A). The decision curve analysis showed an advantage of the base plus LVI model over the base model for the prediction of tumor relapse (Figure 4A). In the base and base plus LVI models, the number of interventions avoided was 33.8 per 100 and 41.3 per 100 patients, respectively, at threshold probability of 45% (the number need to treat: 2.2) (Figure 4B). We could interpret this result to mean that we can reduce unnecessary treatment in 7.5 per 100 patients with a 45% of the risk for tumor relapse.

**TABLE 3:** Development of a risk model

Risk group	Base model (trial eligibility)	LVI model
Low risk	pT0‐2 or ypT0‐1, and pN0	pT0‐2 or ypT0‐1, and pN0 and LVI‐
Intermediate risk		pT0‐2 or ypT0‐1, and pN0 and LVI+
High risk	pT3‐4 or ypT2‐4, or pN+	pT3‐4 or ypT2‐4, and pN0 and LVI‐
Very high risk		pT3‐4 or ypT2‐4, and pN+ or LVI+

Abbreviation: LVI, lymphovascular invasion.

## 4 DISCUSSION

This study investigated the proportion of trial‐eligible patients for adjuvant immunotherapy and its impact on prognosis in localized or locally advanced UC. Because the CheckMate 274 and IMvigor 010 trials included selected patients,^11^, ^12^ we need to recognize the selection biases in those patients to translate the outcomes from clinical trial to practice. We observed that more than half (*n* = 543/1082, 52%) of patients were eligible for adjuvant immunotherapy in our practice. We found a significant difference in DFS and OS between the trial‐eligible and ‐ineligible patients based on the trial inclusion criteria. In addition, DFS and OS were not significantly different between pT0‐2pN0 and ypT0‐1pN0 in the trial‐ineligible patients or between pT3‐4 or pN+ and ypT2‐4 or pN+ in trial‐eligible patients, except for DFS in patients with MIBC (*p* = 0.049). These observations indicate that trial inclusion criteria were feasible for stratifying patients for adjuvant therapy.

Despite their similar inclusion criteria, two pivotal phase III clinical trials (CheckMate 274 and IMvigor 010) showed controversial outcomes.^11^, ^12^ The difference in those trials consist of the types of immunotherapy (programmed death receptor–1, or programmed death receptor‐ligand–1) and the number of patients with UTUC (21% in the CheckMate 274 trial and 7% in the IMvigor 010 trial). The results of the subgroup analysis indicated that both studies showed a potential benefit among patients with advanced disease (pT3‐4 or pN+), urinary bladder tumor, and baseline PD‐L1+ disease. Despite the unfavorable background in the CheckMate274 (i.e., a higher number of patients with UTUC), adjuvant nivolumab therapy resulted in a significant improvement in DFS. The difference between PD‐1 and PD‐L1 might have played some role, but we do not have a clear answer on this point. Accordingly, we speculate that the patients' LVI status may have a key role in this setting because the subanalysis of both clinical trials showed patients with T3‐4 or N+ disease had a tendency of favorable outcomes.^11^, ^12^ Although many studies have suggested the negative impact of LVI+ on prognosis, it was not included in the inclusion criteria in both clinical trials.^13^, ^14^, ^15^, ^16^, ^17^, ^18^, ^19^ We found that more than half of the patients in this cohort had LVI+ or pN+ (very high‐risk) (Figure S4A,D). In addition, when we simply compared LVI status in the trial‐eligible group, the median DFS was significantly worse in patients with LVI+ than in those with LVI−, which was similar to that of patients with pN+ (Figure S4B,C,E,F). Results of the multivariable Cox regression proportional hazards model showed that LVI+ or pN+ were significant factors for poor prognosis. We subsequently developed a risk model that included LVI status (base model plus LVI status: LVI model) and observed a clear difference in prognosis (Figure 3). The LVI model was superior in predicting recurrence, with a c‐index of 0.753 in comparison with the base model (c‐index: 0.699). The number of interventions avoided of 7.5 patients per 100 at the threshold probability (risk of tumor recurrence) of 45% is clinically useful to decrease unnecessary treatment in patients with a marginal status for adjuvant therapy (Figure 4). As the CheckMate 274 and IMvigor 010 trials included patients who had high‐ and very high‐risk, the outcomes might be influenced by the number of patients with very high‐risk. Although there is still no clear reason for the controversial outcomes of both clinical trials for adjuvant immunotherapy, LVI status might play a key role in understanding the difference between the two pivotal trials. Further studies on this issue are necessary.

Several limitations in this study need to be acknowledged. First, because of the retrospective study design, we could not control for selection bias and other unmeasurable confounders. Second, the statistical analysis might be underpowered because of the small sample size. Third, analyses under a single population are a problem for generalization. Also, this was an observational study presenting the well‐known outcomes, and those were not beyond expectations. Nonetheless, this study presents the clinical implications of the eligibility of adjuvant immunotherapy and its impact on prognosis in localized or locally advanced UC. Further studies are required to determine the optimal strategies for the transition from surgical treatment to adjuvant immunotherapy.

## 5 CONCLUSIONS

A total of 52% of patients were potentially eligible for adjuvant immunotherapy. Trial eligibility was significantly associated with a poor prognosis. LVI+ and pN+ may play a key role in the selection of candidates for adjuvant immunotherapy.

## FUNDING INFORMATION

This study was supported by Japan Society for the Promotion of Science (JSPS) KAKENHI (grants 19H05556 [C. O.], 20K09517 [S. H.], 19K18603 [N. F.], 18K16718 [D. N.], and 21K16749 [H. H.]).

## CONFLICT OF INTEREST

The authors have no conflict of interest.

## ETHICS STATEMENT

The present retrospective, multicenter study was performed in accordance with the ethical standards of the Declaration of Helsinki and was approved by the ethics review board of Hirosaki University School of Medicine (authorization No. 2019–099) and all hospitals. Pursuant to the provisions of the ethics committee and the ethics guidelines in Japan, written informed consent is not required for public disclosure of study information in the case of a retrospective and/or observational study using materials, such as existing documents (opt‐out approach).

## AUTHORS CONTRIBUTIONS

Yuki Miura is responsible for data collection. Shingo Hatakeyama is responsible for project development, manuscript editing, data analysis, and data collection. Toshikazu Tanaka, Naoki Fujita, Hirotaka Horiguchi, Yoshiharu Okuyama, Yuta Kojima, Daisuke Noro, Noriko Tokui, Teppei Okamoto, Hayato Yamamoto, Hiroyuki Ito, Takahiro Yoneyama, and Yasuhiro Hashimoto are responsible for the data collection. Chikara Ohyama is responsible project development and critical review.

## Supporting information


**FIGURE S1:** The prognostic impact of pT/pN stage on prognosis in patients with MIBC
**A:** Disease‐free survival in patients with MIBC who were treated with radical cystectomy (RC) alone. **B:** Overall survival in patients with MIBC who were treated with RC alone. **C:** Disease‐free survival in patients with MIBC who were treated with neoadjuvant chemotherapy (NAC) + RC. **D:** Overall survival in patients with MIBC who were treated with NAC + RC.
**Figure S2:** The prognostic impact of pT/pN stage on prognosis in patients with UTUC
**A:** Disease‐free survival in patients with MIBC who were treated with radical nephroureterectomy (RNU) alone. **B:** Overall survival in patients with MIBC who were treated with RNU alone. **C:** Disease‐free survival in patients with MIBC who were treated with neoadjuvant chemotherapy (NAC) + RNU. **D:** Overall survival in patients with MIBC who were treated with NAC + RNU.
**Figure S3:** Prognostic impact of LVI + and pN + on DFS
**A:** Proportion of LVI + or pN + in patients with pT3–4/ypT2–4 or pN + among patients with MIBC. **B:** Disease‐free survival of patients with MIBC between LVI + and LVI − in the trial‐eligible group. **C:** Disease‐free survival of patients with MIBC between pN + and pN − in the trial‐eligible group. **D:** Proportion of LVI + or pN + in patients with pT3–4/ypT2–4 or pN + among patients with UTUC. **E:** Disease‐free survival of patients with UTUC between LVI + and LVI − in the trial‐eligible group. **F:** Disease‐free survival of patients with UTUC between pN + and pN − in the trial‐eligible group.
