# Supplementary material for: Prognostic impact of eligibility for adjuvant immunotherapy in locally advanced urothelial cancer
Source: BJUI Compass. 2021 Oct 8;3(2):146–53. doi: 10.1002/bco2.117 (PMC8988644; doi:10.1002/bco2.117)
Supplement: Supplementary file 1 — FIGURE S1: The prognostic impact of pT/pN stage on prognosis in patients with MIBC A: Disease‐free survival in patients with MIBC who were treated with radical cystectomy (RC) alone. B: Overall survival in patients with MIBC who were treated with RC alone. C: Disease‐free survival in patients with MIBC who were treated with neoadjuvant chemotherapy (NAC) + RC. D: Overall survival in patients with MIBC who were treated with NAC + RC. Figure S2: The prognostic impact of pT/pN stage on prognosis in patients with UTUC A: Disease‐free survival in patients with MIBC who were treated with radical nephroureterectomy (RNU) alone. B: Overall survival in patients with MIBC who were treated with RNU alone. C: Disease‐free survival in patients with MIBC who were treated with neoadjuvant chemotherapy (NAC) + RNU. D: Overall survival in patients with MIBC who were treated with NAC + RNU. Figure S3: Prognostic impact of LVI + and pN + on DFS A: Proportion of LVI + or pN + in patients with pT3–4/ypT2–4 or pN + among patients with MIBC. B: Disease‐free survival of patients with MIBC between LVI + and LVI − in the trial‐eligible group. C: Disease‐free survival of patients with MIBC between pN + and pN − in the trial‐eligible group. D: Proportion of LVI + or pN + in patients with pT3–4/ypT2–4 or pN + among patients with UTUC. E: Disease‐free survival of patients with UTUC between LVI + and LVI − in the trial‐eligible group. F: Disease‐free survival of patients with UTUC between pN + and pN − in the trial‐eligible group. [file BCO2-3-146-s001.pdf]

Fig.S1

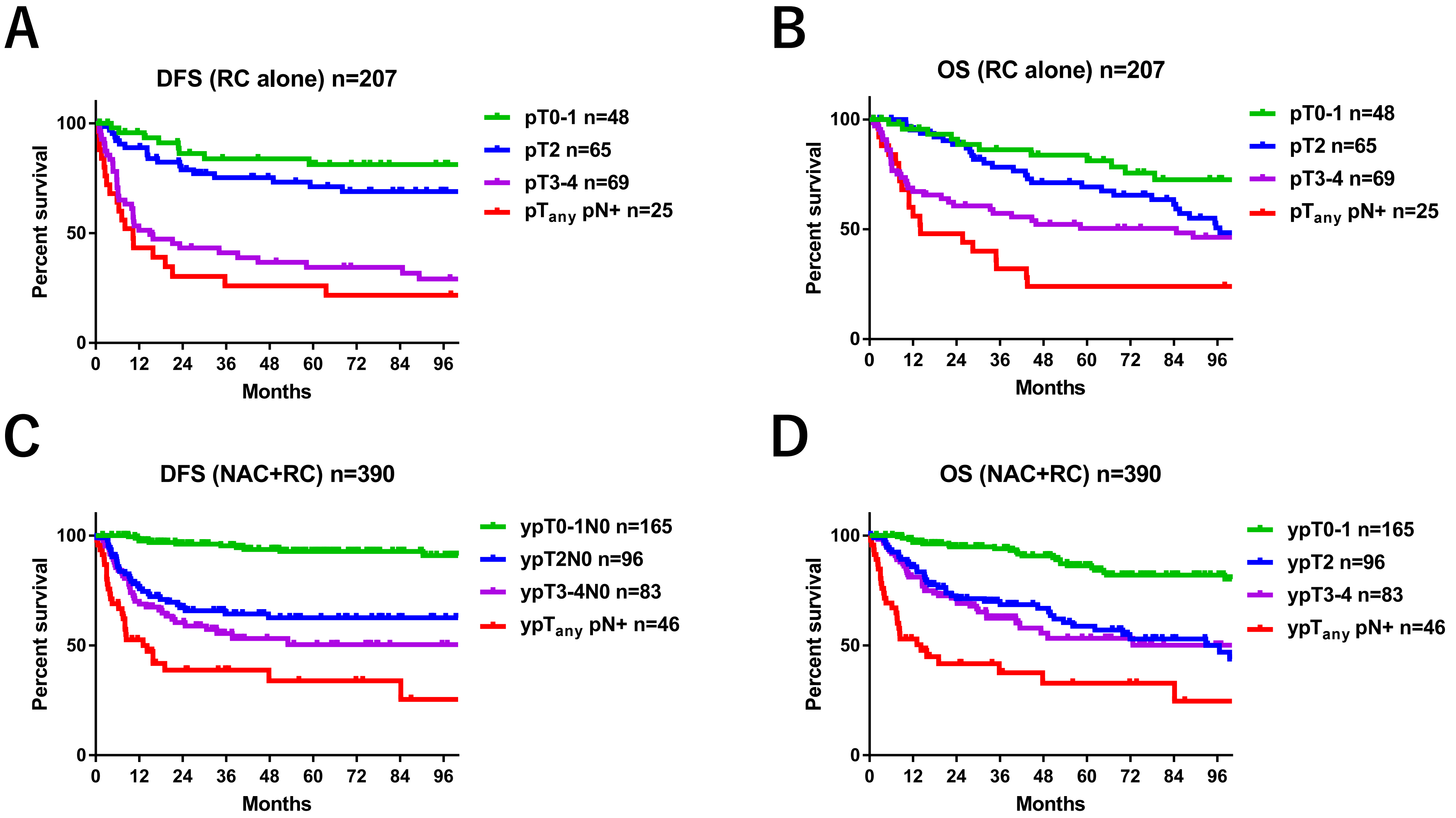
A
DFS (RC alone) n=207
Percent survival
Months
pT0-1 n=48
pT2 n=65
pT3-4 n=69
pTany pN+ n=25
B
OS (RC alone) n=207
Percent survival
Months
pT0-1 n=48
pT2 n=65
pT3-4 n=69
pTany pN+ n=25
C
DFS (NAC+RC) n=390
Percent survival
Months
ypT0-1N0 n=165
ypT2N0 n=96
ypT3-4N0 n=83
ypTany pN+ n=46
D
OS (NAC+RC) n=390
Percent survival
Months
ypT0-1 n=165
ypT2 n=96
ypT3-4 n=83
ypTany pN+ n=46

Fig.S2

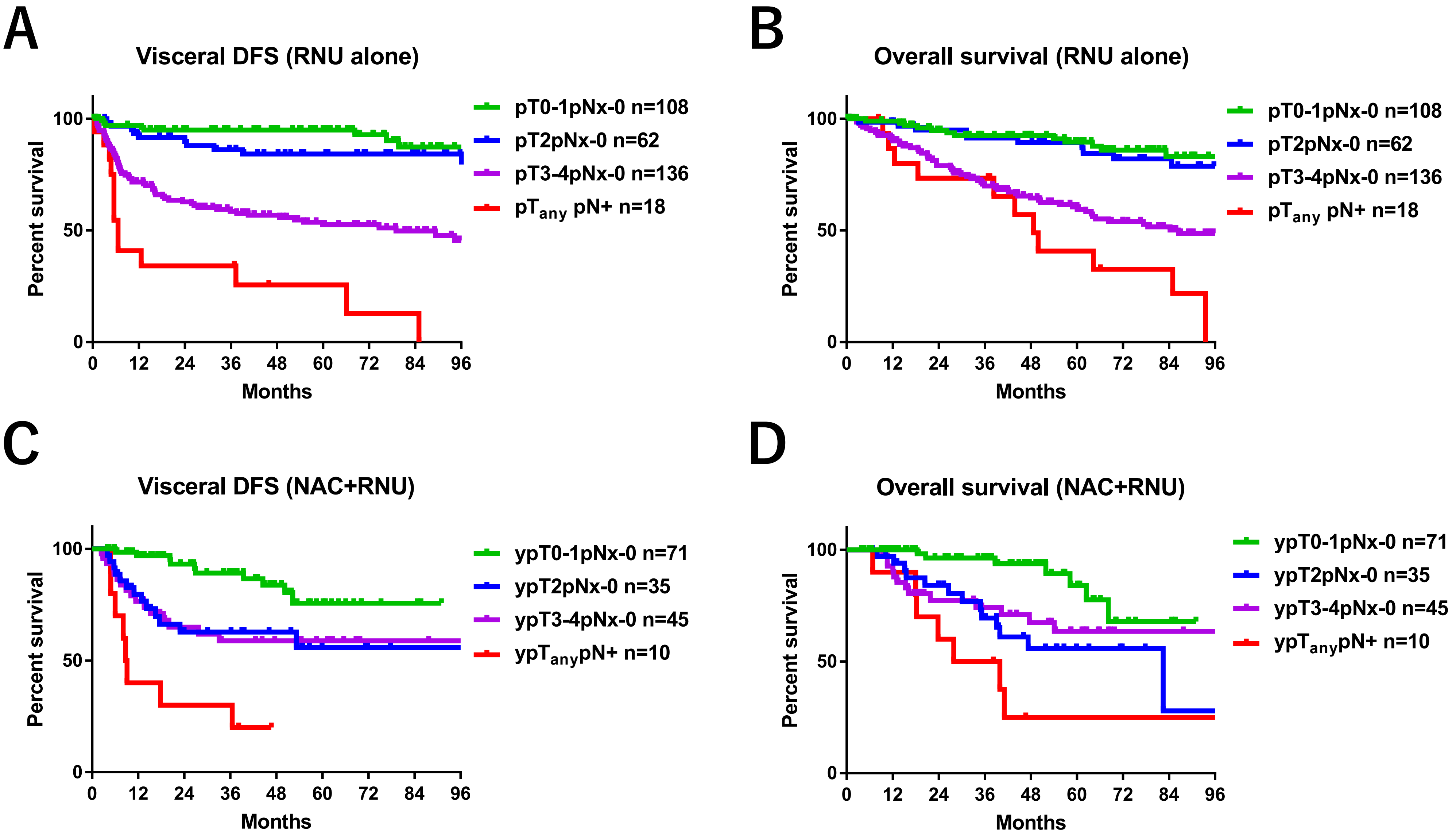

A
Visceral DFS (RNU alone)
pT0-1pNx-0 n=108
pT2pNx-0 n=62
pT3-4pNx-0 n=136
pTany pN+ n=18
Percent survival
Months
B
Overall survival (RNU alone)
pT0-1pNx-0 n=108
pT2pNx-0 n=62
pT3-4pNx-0 n=136
pTany pN+ n=18
Percent survival
Months
C
Visceral DFS (NAC+RNU)
ypT0-1pNx-0 n=71
ypT2pNx-0 n=35
ypT3-4pNx-0 n=45
ypTanypN+ n=10
Percent survival
Months
D
Overall survival (NAC+RNU)
ypT0-1pNx-0 n=71
ypT2pNx-0 n=35
ypT3-4pNx-0 n=45
ypTanypN+ n=10
Percent survival
Months

Fig.S3

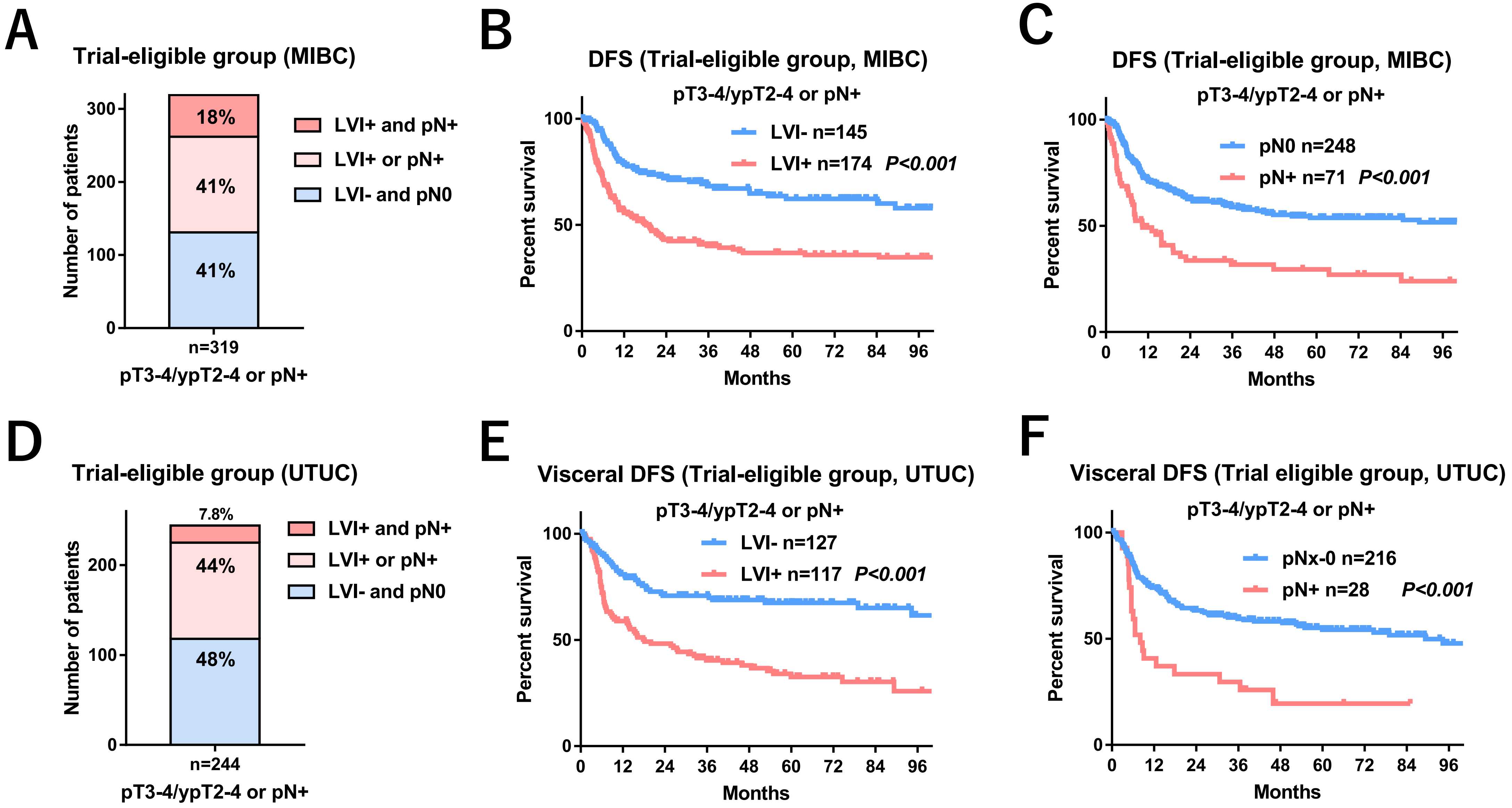

A
Trial-eligible group (MIBC)
Number of patients
300
200
100
0
18%
41%
41%
LVI+ and pN+
LVI+ or pN+
LVI- and pN0
n=319
pT3-4/ypT2-4 or pN+
B
DFS (Trial-eligible group, MIBC)
pT3-4/ypT2-4 or pN+
Percent survival
LVI- n=145
LVI+ n=174 P<0.001
Months
C
DFS (Trial-eligible group, MIBC)
pT3-4/ypT2-4 or pN+
Percent survival
pN0 n=248
pN+ n=71 P<0.001
Months
D
Trial-eligible group (UTUC)
Number of patients
7.8%
44%
48%
LVI+ and pN+
LVI+ or pN+
LVI- and pN0
n=244
pT3-4/ypT2-4 or pN+
E
Visceral DFS (Trial-eligible group, UTUC)
pT3-4/ypT2-4 or pN+
Percent survival
LVI- n=127
LVI+ n=117 P<0.001
Months
F
Visceral DFS (Trial eligible group, UTUC)
pT3-4/ypT2-4 or pN+
Percent survival
pNx-0 n=216
pN+ n=28 P<0.001
Months
0 12 24 36 48 60 72 84 96
